# Effect of surgery on oxidative stress and endogenous tocopherol concentrations in juvenile female dogs

**DOI:** 10.1186/s13028-024-00753-x

**Published:** 2024-07-11

**Authors:** Giovanna Lucrezia Costa, Fabio Leonardi, Patrizia Licata, Marco Tabbì, Nicola Iannelli, Diego Iannelli, Daniele Macrì, Fabio Bruno, Vincenzo Ferrantelli, Vincenzo Nava, Claudia Interlandi, Giuseppe Bruschetta

**Affiliations:** 1 Department of Veterinary Sciences, University of Messina, Via Palatucci Annunziata, 98168 Messina, Italy; 2 Department of Veterinary Science, University of Parma, Via del Taglio 10, 43126 Parma, Italy; 3 Clinica Veterinaria Camagna, Via Fortunato Licandro 13, 84124 VetpartnersReggio Calabria, Italy; 4 Zooprophylactic Institute, Via Gino Marinuzzi 4, 90100 Palermo, Italy

**Keywords:** Analgesia, Dogs, Oxidative stress, Surgery, Tocopherols

## Abstract

**Background:**

Surgery such as ovariectomy causes an inflammatory and oxidative stress. This study was designed to evaluate endogenous tocopherol levels in response to surgical oxidative stress induced by abdominal surgery (ovariectomy) in thirty-two juvenile female dogs. The dogs received meloxicam before surgery (0.2 mg/kg SC) and after surgery (0.1 mg/kg OS every 24 h), 0.03 mg/kg of atropine sulfate (IM), and propofol 4 mg/kg intravenously (IV). General anesthesia was maintained with sevoflurane. Physiological, hematological and biochemical parameters, malondialdehyde (MDA) and α-, δ-, γ-tocopherols were evaluated at baseline, 36 and 48 h after surgery.

**Results:**

The physiological parameters remained within normal ranges. Blood glucose concentration increased, while the albumin levels decreased after surgery. Rescue analgesia was not required. MDA levels increased above the baseline at 36 and 48 h after surgery (P < 0.001). The α-, δ-, and γ-tocopherol concentrations decreased from baseline at 36 and 48 h after surgery (P < 0.001).

**Conclusions:**

Surgery in juvenile female dogs revealed oxidative, increased MDA concentrations, reduced tocopherol levels, and had a clinically insignificant influence on homeostasis.

## Background

Surgery induces inflammatory and oxidative stress [1]. The resulting inflammation, pain and stress induced by surgery can hinder tissue healing and promote the development of post-surgical pathologies such as cancer [1]. Surgical or traumatic injuries, radiation, burns, even from electrosurgery as cauterization, expose the body to infections with consequent release of toxins. Tissue damage leads to hypoxia with consequent activation of polymorphonuclear leukocytes and monocytes, to increase in Reactive Oxygen Species (ROS) levels and to vasoconstriction followed by endothelin-1 release, ischemia and necrosis. The latter are the basis of the development of post-traumatic and post-surgical cancerous lesions. Spinal trauma induces the release of glutamate and ROS generation with consequent progressive loss of neurons. Ischemia and subsequent reperfusion of an organ during surgery (e.g., clamping of the aorta and subsequent release in cardiac, thoracic, and abdominal surgery) cause oxidative and inflammatory stress [1]. Orthopedic surgery is also associated with inflammatory and oxidative stress with higher mortality and morbidity rates in elderly patients compared to young patients [2, 3]. Open orthopedic surgery entails greater involvement of the soft tissues, resulting in greater inflammation that affects the healing process, which may be very slow in open orthopedic surgery [2, 3]. The tourniquet applied to a limb in order to obtain a blood-free operating field causes ischemia. The release of the tourniquet and tissue reperfusion with consequent increase in ROS can induce a delay in tissue healing and predispose to a greater risk of infection [4–6].

Ovariectomy is a commonly performed surgery in dogs for the prevention of pregnancy and for therapeutic purposes such as in cases of pyometra, ovarian and mammary tumors. Surgical stress, pain, hypoxia and reperfusion due to ovarian pedicle ligation are the cause of inflammatory and oxidative stress due to the release of cytokines, prostaglandins and nitric oxide [7]. The anesthetic protocol could be crucial to tissue healing and the progression of pre-existing pathological conditions such as neoplasms [8]. Previous experimental and clinical researches demonstrated that a balanced anesthesia could decrease the adverse reactions due to the surgical stress [8]. The oxidative state can modify various enzyme activities, such as superoxide dismutase (SOD) and catalase (CAT), together with the increasing of the lipid peroxidation mechanisms and inflammation [9, 10]. Malondialdehyde (MDA) is used in biomedical research as an indicator of lipid peroxidation via its reactions with thiobarbituric acid (TBA), resulting in the production of MDA-TBA2, a conjugate which has an absorption peak in the visible spectrum at 532 nm and appears as a reddish-rose hue. This method is useful to detect the lipid peroxidation products, available in the serum samples, that may interact with TBA and absorb light at 532 nm, contributing to the overall absorbance signal measured. The Thiobarbituric acid reactive substances (TBARS) assay is widely accepted as an effective method for measuring oxidative stress levels in biological samples [11]. Its applicability was demonstrated in human serum, low-density lipoproteins, and cell lysates. Biological systems also contain antioxidant enzymes to prevent oxidative tissue damage. When animals are exposed to surgical oxidative stress, they also exhibit a compensatory endogenous antioxidants induction.

The dietary inclusion of vitamin E supplements (tocopherols and tocotrienols) can effectively reduce lipid peroxidation [12, 13]. The strong antioxidant properties of α-tocopherol, δ-tocopherol, and γ-tocopherol make them particularly useful to protect cell membranes [14]. This vitamin also has a crucial preventing role in cardiovascular diseases by minimizing the aggregation of platelets, leading to a reduction in emboli, plaque, and thrombi. Consequently, α-, δ-, and γ-tocopherols are valuable anticoagulants, preventing unwanted clotting of the blood without interfering with the normal aggregation needed to stop bleeding in wounds [15]. The distinctive antioxidant properties of γ-tocopherol, which efficiently converts nitrogen dioxide, a DNA-damaging agent, into nitric oxide, offer a mechanical explanation for its functional role to prevent DNA damage over time [16]. Several studies on cells, animals, and humans revealed that γ-tocopherol can have significant beneficial effects by protecting cells from inflammatory damage [17]. Gamma-tocopherol levels increase in patients anesthetized with propofol, as this molecule has a chemical structure similar to endogenous tocopherols with antioxidant scavenger activity [14]. The physiological regulation, antioxidant mechanism of action, and effect on enzymatic pathways indicate that γ-tocopherol could have a functional role in maintaining the health of the organism [14, 18]. In another study, α-tocopheryl acetate supplementation in the rat diet decreased plasma lipid peroxidation and renewed plasma SOD and GPx activity, which had been impaired in rats on a high-fat diet [19]. Other studies have demonstrated that, α-tocopheryl acetate reduced damage-induced ischemia/reperfusion ROS production in mice and prevented oxidative modifications of phospholipids [20]. In a previous investigation, it was observed that α-tocopheryl acetate supplementation reduced the serum concentration and the MDA mammary muscle content in broiler chickens fed with a linseed oil-enriched diet [21, 22].

The aim of the present study was to assess the concentration of endogenous tocopherols in response to inflammatory and oxidative stress induced by abdominal surgery (ovariectomy) in juvenile female dogs.

## Methods

In order to adequately determine the sample size for “a priori” X^2^ tests (goodness-of-fit tests: contingency tables), G Power 3.1 software was used, with an effect size (f) of 0.5, a significance level (α) of 0.05, a power (1-β) of 0.80 and only one group. Thirty-two female juvenile dogs, crossbreeds, 1 year old ± 1.5 months and weighing 16 ± 0.5 kg. The inclusion criterion of the patients was to undergo ovariectomy as elective surgery [23]. The criterion, which precluded the exclusion of a subject in the study, was alterations of blood count and biochemical parameters.

### Treatment administration and anesthesia

During the study, the dogs were fed the same monoprotein feed added with 235 mg/kg of vitamin E (Vet Line, Italy). Dry food once a day and water “ad libitum” were given up to eight hours before the start of surgery. The dogs received 0.2 mg/kg of meloxicam subcutaneously (SC) (Metacam 2% Boehringer Ingelheim Italia S.p.A.) and 0.03 mg/kg of atropine sulfate intramuscularly (IM) (atropine sulfate 0.1% A.T.I.). A catheter of 20G × 32 mm (DELTA VEN) was placed in the cephalic vein for the administration of 5 ml/kg/h of lactate ringer solution for the duration of the surgery. Twenty minutes after premedication, anesthesia was induced with 4 mg/kg of propofol (Proposure 1%, Merial, Assago, Italy) intravenously (IV). Intubation was carried out by a Magill cuffed tube. General anesthesia was maintained with sevoflurane (Sevoflo Zoetis Italy) supplied by 100% oxygen using a rebreathing circle system. Ventilation was performed using a pressuremeter ventilator (SIMV) (GE Datex-Ohmeda Avance Ultramed Italy) and respiratory parameters were set as follows: respiratory rate 12 breaths min^−1^, positive pressure at the end of expiration (PEEP) 4 cm H_2_O, inspiratory/expiratory ratio (I: E) 1:7, and airway pressure 12 cm H_2_O. After surgery, 0.1 mg/kg of meloxicam was administered orally (OS) every 24 h.

### Physiological and anesthetic parameters

Heart rate (HR, beats per minute) measured by auscultation using a stethoscope (Littmann^®^ USA), respiratory rate (RR, acts per minute) measured by counting thoracic wall excursions, non-invasive blood pressure (mmHg) (systolic, SAP; mean, MAP; diastolic, DAP) measured by placing a small-sized cuff around the tail base, body temperature (T, °C), end-tidal carbon dioxide tension (EtCO_2_, mmHg), arterial hemoglobin oxygen saturation (SpO_2_, %), and the inspired and expired sevoflurane concentration (CSI/CSE) were measured using a monitor (GE Datex-Ohmeda Avance multiparametric monitor for anesthesia Ultramed Italy). These parameters were recorded at T_0_ (baseline), after a 30 min acclimation period in the surgical preparation room, at 20 min after premedication (except EtCO_2_ and CSI/CSE), after the induction of general anesthesia, at skin incision, at laparotomy, during traction and the removal of the first ovary, during traction and the removal of the second ovary, and at skin suture.

### Assessment of intra- and postoperative response to surgical stimulus

The evaluation of intraoperative response to the surgical stimulus was evaluated using a cumulative pain scale [24]. Briefly, a numerical score between 0 and 4 was assigned based on the percent change from RR, HR, and SAP values recorded after the induction of general anesthesia (A) throughout the surgery according to the following scheme: 0 = variation ≤ 0%; 1 = variation ≤ 10%; 2 = variation > 10% but ≤ 20%; 3 = variation > 20% but ≤ 30%; and 4 = variation > 30%. The sum of the scores for the three parameters was the response to the surgical stimulus. A total score of 10, corresponding to the 20% increase in (HR, RR and SAP), was considered the cut-off point for the rescue analgesia administration represented by 2 μg/kg of fentanyl (Fentadon, Dechra). A postoperative pain score was assigned, from recovery to 24 h after surgery every 6 h, using the canine acute pain scale (Colorado State University Veterinary Medical Center) with scores 0–4. Score 2, corresponding to moderate to mild pain, was the cut-off point for the administration of postoperative rescue analgesia represented by 0.2 mg/kg of methadone (Semfortan, Dechra) IM.

### Hematological, biochemical parameters and oxidative stress

After carrying out the aforementioned measurements, 5 ml of blood was drawn from the cephalic vein. The same operator took all the samples. Each sample was divided into two aliquots, of which, one was placed in a vacuum serum isolation tube (serum clot activator Z, Vacuette^®^, Greiner Bio-One, Kremsmünster, Austria) used to determine the biochemical parameters (glycemia; aspartate transaminase, AST; alanine aminotransferase, ALT; total protein; albumin; BUN). Furthermore, a blood sample was used for oxidative stress evaluation by gaging lipid peroxidation, catalase (CAT), superoxide dismutase (SOD), myeloperoxidase (MPO), butyrylcholinesterase (BuChe) and tocopherols (α-tocopherol, δ-tocopherol and γ-tocopherol); another aliquot of blood was placed in a vacuum tube with EDTA (K3-EDTA, Vacuette^®^, Greiner Bio-One, Kremsmünster, Austria) to perform blood count (only at the baseline). Both tube groups were immediately cooled at 4 °C and subsequently (within 3–4 h) centrifuged (only the sample placed in the serum tube) for 15 min at 1500 g to obtain the serum aliquot. Complete blood count was assessed by IDEXX Italy. Glucose, albumin and total proteins were measured using the glucose oxidase/peroxidase, bromocresol green, and biuret methods, respectively. AST and ALT were measured at 37 °C using kinetic methods. To evaluate all of the above parameters, a UV–Vis spectrophotometer (A560, Fulltech, Rome, Italy) was used [25, 26]. For biochemical assessment, blood samples were collected at baseline and 36 h after surgery. For oxidative status evaluation, blood samples were taken at baseline, 36 and 48 h after surgery.

### Determination of CAT, SOD, MPO and BuChE

CAT activity was determined through an enzyme reaction in the presence of H_2_O_2_. Prior to starting the assay, all reagents were equilibrated to room temperature. The final test volume was 240 μL in each well. The samples and standards were assayed in duplicate, utilizing catalase sample buffer (1X) and diluted assay buffer. Measurements of absorbance were taken at 540 nm using a BIORAD 680 microplate reader (BIORAD Laboratories, Italy) [27]. SODs are metalloenzymes that catalyze the decomposition of the superoxide anion into molecular oxygen and hydrogen peroxide. The final volume for each duplicate well was determined to be 230 μL. All reagents were used at 25 °C prior to start the test, except for xanthine oxidase. Absorbance was monitored between 440–460 nm using the BIORAD 680 plate reader (BIORAD Laboratories, Italy) [28]. MPO activity was measured using the dianisidine-H70 method, for 96-well plate. Samples were added in triplicate to a mixture consisting of 0.53 mM o-dianisidine hydrochloride, 0.15 mM hydrogen peroxide and 50 mM potassium phosphate buffer (pH 6.0). After a 5 min incubation at 25 °C, the reaction was arrested with 30% sodium azide and the absorbance measured at 460 nm (BIORAD 680 plate reader, BIORAD Laboratories, Italy) [29]. The BuChE assay involves the substrate hydrolysis and thiocholine formation, which reacts with 2-nitrobenzoic acid dissolved in 625 µL of BuChE Assay Buffer. Absorbance was instantly measured at 412 nm 25 °C with a BIORAD 680 plate reader (BIORAD Laboratories, Italy) [30].

### Determination of Malondialdehyde

The phosphoric acid (85%, 15 mol/L), sodium hydroxide, SDS (8.1%), and sodium chloride were bought from Merck (Darmstadt, Germany). The thiobarbituric acid (TBA) was purchased from Fluka (Buchs, Switzerland). All of the reagents were of analytical grade or the highest grade available. The malondialdehyde (MDA) standard was prepared through the hydrolysis of TMP; the TBA reagent (0.11 mol/L: 800 mg TBA dissolved in 50 mL, 0.1 mol/L NaOH) was prepared for the assay. For the quantitative determination of TBARS, 200 μL of an MDA standard solution were used instead of plasma. MDA stock solutions were prepared through the hydrolysis of 50 μL of TMP (10 mmol/L) in 10 mL 0.01 M hydrochloric acid for 10 min at room temperature. The MDA stock solution was diluted with ultrapure water to different concentrations of MDA standards. Calibration in the plasma was achieved by adding phosphoric acid containing different amounts of MDA to the pooled samples of plasma. After adding the samples (200 μL) to the reaction mixture, the tubes were incubated at 90 °C in a water bath. Lipid peroxidation in the blood serum samples was evaluated by measuring the production of MDA after reacting with glacial acetic acid thiobarbituric acid for 1 h. The tubes were then placed into ice to arrest the reaction. After cooling to room temperature, 100 μL of standards and samples were placed into a flat-bottom 96-well multititer plate. Absorption was read at 535 nm and 572 nm to correct for baseline absorption in a plate reader. MDA equivalents (TBARS) were calculated using the difference in absorption at the two wavelengths, and quantification was determined with the aid of calibration curves.

### Determination of α-tocopherol, δ-tocopherol, and γ-tocopherol

N-hexane and ethyl acetate were UHPLC/MS grade, purchased from Optima, Fisher Chemical products (Milan, Italy). Alpha-tocopherol, δ-tocopherol, and γ-tocopherol together with the solvents were purchased from Sigma-Aldrich (Milan, Italy). Blood samples were purchased from dogs via venipuncture and centrifuged (2000 g, 8 min at 4 °C). Into an amber microcentrifuge tube, the serum was separated, aliquoted and frozen at − 70 °C. Then the samples were thawed at room temperature, shaken slowly by inversion and centrifuged at 448 g at 4 °C for 15 min. Serum samples of 200 μL were deproteinated by adding 200 μL of ethyl alcohol with 0.5 mol/L echinenone and 4 mol/L tocol in amber glass vials. Vortex was used to mix the samples for about 3 min, then 800 μL of hexane were added and mixed for 10 min at 252 g with a mechanical vortex. Then the sample was centrifuged for 15 min at 287 g and the respective supernatants were placed in amber glass vials. The extraction with hexane was replicated. The supernatants were evaporated under nitrogen stream at room temperature. The residues were reconstituted in 100 L mobile phase containing 30 mg/L BHT without ammonium acetate followed by 2 min vortex and 2 min ultrasonic bath. Twenty μL of sample were introduced into an HPLC system. The resulting residue and injectable solvent remained stable for up to 24 h at 4 °C. A Shimadzu (Milan, Italy) LC-20AD HPLC system with RF-20A fluorescence detector, a CBM-20A controller, a CTO-20A column oven, an LC-20AD pump and a DGU-20A3 degasser was used to perform chromatographic analysis. The analyses were carried out using a LiChrosorb^®^ Si 60 (5 μm) column (4.6 mm I.D. × 250 mm), which was preserved by a guard column with the same stationary phase. The analyses were carried out at 50 °C under isocratic conditions using a mobile phase made from n-hexane/ethyl acetate (90:10 v/v). The injection volume was 15 μL and the flow rate was 0.9 mL/minute. The fluorescence excitation and emission wavelengths were 295 nm and 330 nm, respectively. Alpha-, δ- and γ-tocopherols were determined using commercial standards, the quantitative analysis was carried out using the external standard method with appropriate calibration curves, and all quantification was calculated as the average value of three repeated measurements. Results were expressed as mean ± SEM.

### Statistical analysis

Statistical analysis was carried out with SPSS version 27.1 (IBM Italy). The data were analyzed for normality using the Shapiro–Wilk test and described as mean ± SEM. Differences along the timeline were analyzed with a t-test or the Wilcoxon test as appropriate. Inter-observer agreement for the quality of postoperative analgesia was analyzed using Kendall’s coefficient of concordance W. Multiple linear regression between MDA and tocopherols was performed. The differences were considered significant at P ≤ 0.05.

## Results

The total number of subjects recruited in the present study was 32, effective statistical power of the sample is 0.80. Data, except for the temperature, were not normally distributed. The level of agreement among the observers who assigned the postoperative pain scores was high (W = 1). All selected patients completed the study.

Physiological parameters remained within the normal range for patients under general anesthesia. SPO_2_ was 96/100%, showing good tissue oxygenation. ETCO_2_ decreased along the timeline from 45 to 36/38 mmHg, showing good adaptation of the patient to the pressure metric ventilator and a good anesthetic plan. The inspired and expired concentrations of sevoflurane were 5–3% CSI and 4–7% CSE, respectively (Table 1). The CPS scores were 0–6 for the entire duration of the surgery (Table 2). The canine acute pain scale score assigned was 0 for the entire postoperative period. No patient required either intraoperative or postoperative rescue analgesia. The blood count was normal for all subjects. Glycemia increased at 36 h after surgery compared to baseline (P = 0.007). Albumin decreased at 36 h after surgery compared to baseline (P = 0.008). Aspartate transaminase (AST) (P = 1.000), alanine aminotransferase (ALT) (P = 1.000), total protein (P = 0.371) and blood urea nitrogen (P = 1.000) were normal at 36 h after surgery as the baseline (Table 3). MDA levels were significantly higher at 36 h and 48 h after surgery, compared to baseline values (P < 0.001). Alpha-tocopherol (P < 0.05), δ-, and γ-tocopherol concentrations were significantly lower at 36 h after surgery compared to baseline values (P < 0.001). Multiple linear regression between the concentration of MDA and tocopherols showed a significant reduction in the latter at 36 and 48 h after surgery (P < 0.001) (Table 4). CAT, SOD, MPO and BuChE levels did not show any significant change (P > 0.05) (Table 4).

**Table 1:** Physiological and anesthetic parameters

	Baseline	Premedication	Anesthesia	Skin incision	Laparotomy	Pedicle traction I	Pedicle traction II	Skin suture
HR (Beats min)	121 ± 3	130 ± 6	147 ± 2	152 ± 4	141 ± 2	144 ± 4	145 ± 4	137 ± 3
RR (Breaths min)	40 ± 2	55 ± 6	12 ± 0	12 ± 0	12 ± 0	12 ± 0	12 ± 0	12 ± 0
SAP (mmHg)	125 ± 3	108 ± 3	102 ± 1	106 ± 3	116 ± 1	114 ± 3	113 ± 3	109 ± 3
MAP (mmHg)	107 ± 8	101 ± 12	101 ± 4	107 ± 10	109 ± 2	110 ± 9	110 ± 9	105 ± 9
DAP (mmHg)	60 ± 1	57 ± 9	52 ± 1	57 ± 6	62 ± 1	58 ± 1	57 ± 5	57 ± 6
EtCO2 (mmHg)			43 ± 2	38 ± 1	36 ± 1	36 ± 1	37 ± 0	36 ± 1
SpO2 (%)			98 ± 0.25	98 ± 0.15	98 ± 0.22	99 ± 0.15	99 ± 0.35	98 ± 0.31
T (°C)	39 ± 0.06		39 ± 0.06	39 ± 0.01	38 ± 0.00	38 ± 0.06	38 ± 0.06	38 ± 0.2
CSI (%)			5 ± 0.17	5 ± 0.04	5 ± 0.09	4 ± 0.16	3 ± 0.36	4 ± 0.26
CSE (%)			4 ± 0.11	4 ± 0.12	4 ± 0.09	4 ± 0.12	3 ± 0.31	3 ± 0.21

20 min after the administration of atropine and meloxicam, after the induction of general anesthesia, at skin incision, laparotomy, first ovarian pedicle traction, second ovarian pedicle traction, and skin suture. The values are expressed as mean ± SEM (HR, RR, SAP, EtCO_2_, and SPO_2_)

*HR* heart rate, *RR* respiratory rate, *SAP* systolic arterial blood pressure, *DAP* diastolic arterial blood pressure, *MAP* mean arterial blood pressure, *EtCO*_*2*_ end-tidal carbon dioxide tension, *SpO*_*2*_ arterial hemoglobin oxygen saturation, *T °C* body temperature, *CSI%* CSE% concentration of inspired and expired sevoflurane at baseline

**Table 2:** Intraoperative response to surgical stimulus score

Cumulative pain score				
Skin incision	Laparotomy	Pedicle traction *I*	Pedicle traction *II*	Skin suture
2 (0/5)	1.5 (0/3)	1.8 (0/6)	1.6 (0/6)	1.45 (0/4)
2 ± 0.3	1.5 ± 0.2	1.8 ± 0.4	1.65 ± 0.5	1.45 ± 0.3

*CPS* cumulative pain score, by giving scores of percent variations, compared with the values recorded after induction of general anesthesia of heart rate, respiratory rate, systolic arterial blood pressure, according to the following scheme: 0 = variation ≤ 0%; 1 = variation ≤ 10%; 2 = variation > 10% but ≤ 20%; 3 = variation > 20% but ≤ 30%; and 4 = variation > 30%. The sum of the scores gives us the total score. The values are expressed as mean ± SEM and median (range)

**Table 3:** Biochemical parameters at baseline and 36 h after surgery

	Baseline	36 h after surgery	P value
Blood glucose (mg/dL)	88 ± 1.47	129 ± 6.94	**0.007**
ALT (U/L)	18 ± 1.63	18 ± 1.63	**1.000**
AST (U/L)	27 ± 7.23	27 ± 7.54	**1.000**
Total Protein (g/dL)	7 ± 0.60	7 ± 0.81	**0.371**
Albumin (g/dL)	5 ± 0.35	4 ± 0.18	**0.008**
BUN (mg/dL)	18 ± 1.30	18 ± 1.30	**1.000**

The values are expressed as mean ± SEM

*ALT* Alanine Aminotransferase, *AST* Aspartate Transaminase, *BUN* Blood Urea Nitrogen

**Table 4:** Values measured in blood at baseline, 36 h and 48 h after surgery

	Baseline	36 h after surgery	48 h after surgery	P value
MDA (μg\mL)	35 ± 6.92	56 ± 8.85	55 ± 8.77	** < 0.001**
α-tocopherols (mg/L)	13.4 ± 1.41	12.73 ± 1.45	12.6 ± 1.5	** < 0.05**
γ-tocopherols (mg/L)	4 ± 0.28	2.88 ± 0.08	2.85 ± 0.07	** < 0.001**
δ-tocopherols (mg/L)	1.2 ± 0.86	0.85 ± 0.07	0.83 ± 0.05	** < 0.001**
CAT (U/mL)	5.91 ± 0.038	5.03 ± 0.082	7.84 ± 0.15	**0.7**
SOD (U/mL)	6.32 ± 0.125	6.32 ± 0.125	6.32 ± 0.125	** > 0.05**
MPO (U/mL)	1.48 ± 0.075	1.35 ± 0.095	1.31 ± 0.131	** > 0.05**
BuChE (U/mL)	0.505 ± 0.085	0.638 ± 0.136	0.553 ± 0.147	** > 0.05**

The values are expressed as mean ± SEM

*MDA* Malondialdehyde, *CAT* α-tocopherol, δ-tocopherol, γ-tocopherol, catalase, *SOD* superoxide dismutase, *MPO* myeloperoxidase, *BuChe* butyrylcholinesterase at baseline, 36 h and 48 h after surgery

## Discussion

Ovariectomy performed under general anesthesia with a balanced anesthetic protocol including perioperative meloxicam, propofol, and sevoflurane, in juvenile female dogs, resulted in an increased MDA and decreased tocopherols at 36 and 48 h after surgery. The aforementioned drugs and their combination are commonly used in anesthesia and for pain management in dogs and humans [31–34]. Meloxicam is an oxicam derivative and it is included in the non-steroidal anti-inflammatory drugs class (NSAIDs). It has anti-inflammatory and analgesic properties due to its selective inhibition of the inducible isoform (COX-2) of the enzyme cyclooxygenase. Previous studies in dogs have demonstrated that meloxicam provides good perioperative analgesic efficacy at doses of 0.2–0.1 mg/kg and does not cause significant changes in physiological parameters [7]. Propofol is an injectable anesthetic usually employed for induction and maintenance of general anesthesia in dogs [32–34]. Due to its chemical similarity to vitamin E, antioxidant capabilities of propofol are outstanding [14, 35, 36]. Sevoflurane is a commonly used halogenated anesthetic in clinical settings due to its low potential for cardiotoxicity, minimal airway irritation, low solubility coefficient with plasma proteins, neuroprotective and anti-inflammatory properties [37]. Furthermore, its rapid clearance through the respiratory system facilitates a rapid postoperative recovery. The anesthetic protocol used in the present study proved to be effective and free of clinically diagnosable side effects. Physiological parameters remained to be in the physiological ranges for dogs under general anesthesia. ETCO_2_ demonstrated good patient response to the pressure metric ventilator, confirming the successful anesthetic plan. This aspect is crucial for managing the anesthetized patient as a rapid achievement of the anesthetic level suitable for surgery shortens operating times and improves analgesic efficacy due to an immediate synergistic effect between halogenated anesthetic and NSAIDs. This synergistic action between halogenated and NSAIDs is due to low solubility of sevoflurane in the blood and rapid elimination from the respiratory system [37]. The CPS scores remained between 0 to 6 throughout the surgery, with an assigned value of zero for the entire postoperative period. No patients required either intraoperative or postoperative rescue analgesia. However, although perioperative pain management was clinically optimal, the surgery resulted in inflammatory and oxidative stress, as demonstrated by decreased albumin levels, increased glycaemia and serum MDA concentration. Multiple linear regression between the concentration of MDA and tocopherols revealed a reduction in the latter at 36 and 48 h after surgery. The concentration of albumin in the blood may be another clinical indicator of the patient's well-being during and after the surgery. As inflammation enhances capillary permeability, postoperative inflammation may result in hypoalbuminemia. In our study, albumins were significantly lower compared to baseline, but within physiological ranges. However, the subjects involved in this study were juvenile patients and therefore, their metabolic rate and sensory and neuronal responses may differ from those observed in adult animals [38]. In general, prevention or management of hypoalbuminemia in healthy surgical patients is recommended rather than attempting to correct it [39]. Hyperglycemia predisposes to multiple postoperative complications. Nevertheless, perioperative glycemia assessments is not a common clinical practice. A clinical trial in humans showed that healthy patients who experienced an increase in blood glucose after major surgery did not have postoperative complications, whereas diabetic patients reported complications [38, 40]. Surgery causes inflammation and therefore oxidative stress independently of perioperative pain management. During surgery, the organ undergoes transient ischaemia and reperfusion processes resulting in oxidative reactions. Due to the significant impact of oxidative stress on post-surgical pathologies, recent research has focused on identifying substances that can prevent the development of this inflammatory state during surgery [1]. Several studies demonstrated that there is an increase in stress after surgery, such as ovariectomy in dogs [41]. There is some information on the evaluation of ovariectomy inflammatory and oxidative stress. In fact, many researches limit themselves to evaluate and compare different ovariectomy surgical techniques, without carrying out an objective evaluations, toxicological and biochemical, such as determination of plasmatic concentration of MDA and other oxidative parameters, glycemia and albuminemia [42–46]. Previous reports found, that in spayed female dogs, plasma concentrations of thiobarbituric acid-reactive substances (TBARS) were increased after the thirtieth day postoperatively [41]. The determination of this oxidative status certainly represented a method of prevention for various pathologies that could arise from ovariectomy in dogs [41].

Lipids represent a major erythrocyte membrane component susceptible to free radical attack (lipid peroxidation), which promotes alterations to the integrity and membrane shape, undermining the cell functions. Malondialdehyde (MDA) is a substance produced as a result of lipid peroxidation, and its levels indicate the degree of oxidative stress produced [47]. The redox state can modify the functioning of numerous enzymes, including superoxide dismutase (SOD) and catalase (CAT). Also the redox state can boost lipid peroxidation mechanisms [10, 11]. Nevertheless, our study didn’t detect significant changes in these enzymes [48]. Alpha-tocopherol is the preferred form of vitamin E and it accumulates in blood and tissues. Alpha-tocopherol acts as a crucial antioxidant defence against lipid peroxidation in all mammals and its primary function involves the protection of polyunsaturated fatty acid (PUFA) membrane against oxygen radicals. It inhibits the cellular and mitochondrial membrane lipid peroxidation, by scavenging lipid peroxyl radicals, which are subsequently converted to tocopheroxyl radical [9, 19]. The dietary tocopherol supplementation may decrease the susceptibility to lipid peroxidation in the tissues of dogs undergoing surgery [49]. Therefore, tocopherols administration could have a protective effect against the oxidative stress, observed in this study as an increase of serum MDA. However, it is important to emphasize that antioxidants can have a pro-oxidant action when they exceed the normal physiological tissue concentration. Thus, vitamin E supplementation has to be preconized with caution [50]. In our study the plasma concentrations of α-tocopherol, δ-tocopherol and γ-tocopherol were reduced after surgery, remaining within the physiological ranges referred to humans (5.5 to 17.0 mg/L), since there are no reference values for the concentration of plasma tocopherols in dogs [51]. This result could be associated with the effective anesthetic management of the patients undergoing surgery, as oxidative markers. Therefore, incorporating tocopherol measurements into the surgical patient evaluation could be useful as a screening tool and, consequently, it could become an integral part of the perioperative surgery assessment. The ability of tocopherols to reduce oxidative stress was confirmed in various studies [14, 19, 52]. Some researchers have reported that, tocopherols improved clinical symptoms, reduced free radical and pro-inflammatory cytokine synthesis in human patients with osteoarthritis [14, 19]. In dogs with osteoarthritis, the same effects were observed [52]. Moreover, tocopherol integration in the early stages of surgically induced osteoarthritis in dogs reduces pain, the production of pro-inflammatory markers (PGE_2_, NOx) in the synovial fluid, and the histological lesions of the articular cartilage [14, 19, 52]. In particular, α-tocopherol may modulate various cellular functions beyond its antioxidant properties [52].

## Conclusions

Abdominal surgery in juvenile female dogs revealed inflammatory and oxidative stress evidenced by increased MDA concentrations and reduced tocopherol levels, which had a clinically insignificant influence on homeostasis.

## Data Availability

The datasets used in the current study are available from the corresponding author on reasonable request.

## Declarations

### Prior publication

Data have not been pu previously.
